# The effect of translation on the binding energy for transition-metal porphyrines adsorbed on Ag(111) surface

**DOI:** 10.3762/bjnano.10.70

**Published:** 2019-03-13

**Authors:** Luiza Buimaga-Iarinca, Cristian Morari

**Affiliations:** 1 National Institute for Research and Development of Isotopic and Molecular Technologies,67-103 Donat, 400293 Cluj-Napoca, Romania

**Keywords:** Ag(111) surface, DFT+U, metal porphyrine, van der Waals

## Abstract

The characteristics of interaction between six transition-metal porphyrines and the Ag(111) surface are detailed here as resulted from DFT calculations. Van der Waals interactions as well as the strong correlation in 3d orbitals of transition metals were taken into account in all calculations, including the structural relaxation. For each system we investigate four relative positions of the metallic atom on top the surface. We show that the interaction between the transition metal and silver is the result of a combination between the dispersion interaction, charge transfer and weak chemical interaction. The detailed analysis of the physical properties, such as dipolar and magnetic moments and the molecule–surface charge transfer, analyzed for different geometric configurations allows us to propose qualitative models, relevant for the understanding of the self-assembly processes and related phenomena.

## Introduction

Metalloporphyrins are organo-metallic compounds exhibiting a wide range of optoelectronic, magnetic, and mechanical properties that are interesting for nanotechnology applications ([1–2]. The core of these compounds is porphyrin, a planar heterocycle, formed by four pyrrole moieties [2]. The π-system of porphyrin contains 18 electrons along the shortest cycle path. Consequently, the 4*n* + 2 Hückel rule is expected to play a key role in all their properties [2].

The applications of porphyrins in nanotechnology are related mainly to the structures formed after deposition on various substrates. A long list of applications can be found in the literature, ranging from molecular sensors [3] over memory devices [4] to light-harvesting structures [5–7]. Among all porphyrin compounds, transition-metal porphyrins (TMPPs) are of particularly interest. Because they accommodate a transition-metal atom in the center, the tuning of spin states is possible by chemically substituting the central atom. Consequently, detailed investigations of the molecular interactions between TMPPs and metal surfaces has received extensive experimental [3–23] and theoretical [24–26] attention.

Among the coinage metals typically used for applications, Ag(111) has attracted particular interest [27–29]. A complex behavior of the porphyrins on this surface was shown. Parallel orientation with respect to the surface is preferred for sub-monolayer coverage, while multilayer coverage leads to a tilted orientation [27]. At 0.88 ML coverage the presence of ordered structures was distinct with reactive internal coupling occurring at 573 K [30–31]. It is believed that the metal center has typically only little influence on the self-assembly structures formed by TMPPs. This is a consequence of the fact that self-organization is dominated by the periphery of the porphyrin [32–38]. As an example, we note the case of mixtures of Co-TPP and Ni-TPP (TPP = tetraphenylporphyrin) on Au(111) forming ordered islands with random distributions of the two metallic centers [28].

While the main adsorption forces are of physical nature, covalent and coordinative bonds can be formed between the transition metal and the underlying metallic slab [26,39–40]. These bonds between a localized state (transition-metal atom) and the energy band in the surface are asking for complex models in order to be fully understood. In [26], the substrate has been treated as an additional ligand. By attaching this cluster to the metal center, a model for the formation of molecule–surface bonds was proposed. Yet, the band character of the metallic substrate is partially lost in this model. The chemisorptive contributions to the TMPP–metal binding energy may lead to a charge transfer between molecule and surface [41]. This constitutes a local perturbation for electronic, structural [42–43] and magnetic [44–45] properties of the adsorbate as well as of the substrate. Such modifications of the electronic structure are of great interest for all potential applications. Theoretical assessment of the adsorption mechanism of TMPP on silver in the framework of DFT ask for an accurate estimation of the van der Waals dispersive interactions, which are expected to be important in the process. Following the increasing awareness of this issue, various methods have been proposed [46–47], many of them being successfully applied the study of the adsorption of porphyrins on metals [26,48–50]. Our approach is based on one newly developed exchange–correlation functional including the van der Waals effect [51], rather than on the Grimme corrections. In order to catch the effect of strong electronic correlation specific to localized orbitals (such as 3d orbitals of transition metals in TMPPs) we included DFT+U corrections in our calculations. Our aim is to describe the dependence of physical parameters such as binding energy, dipolar moment and charge transfer on the transition-metak atom in the TMPP as well as on the position of the molecule on top of the silver surface. These data will provide a base for deeper understanding of the self-assembly processes involving TMPP and their derivatives on noble-metal surfaces.

## Computational Details

The computational setup and calculations performed for the present study were similar to those used in our study on metal phthalocyanines adsorbed on a gold surface [29]. We used the “Siesta” code [52–54]. One main characteristic of “Siesta” is the use of norm-conserving pseudopotentials [55] while the molecular states are represented as linear combinations of atomic orbitals (LCAO). We used the van der Waals exchange–correlation functional vdW-DF-cx of Berland and Hyldgaard (BH) [51]. The BH functional has been proven to be well suited for the study of bulk metals as well as for adsorption of aromatic molecules on Ag(111) [56]. According to the conclusion of the abovementioned study, the vdW-DF-cx functional is capable of accurately describing the structure and properties of a wide range of systems ranging from bulk oxides, to molecules adsorbed at surfaces and in porous media. In particular the vdW-DF-cx functional can reliably describe both the molecular adhesion on silver and the Ag bulk structure [56]. A correct geometric model for both molecule and metallic slab represents a major advantage in the study of molecular adsorption as previously pointed out [57].

All six systems investigated by us were confined to a unit cell that allows the study of a periodical Ag(111) surface. It has a size of 7 × 7 Ag atoms and includes four atomic layers, with a total of 196 silver atoms. The metal surface was set to be parallel to the *XOY*-plane, while the length of the cell along the *OZ*-axis was *L_Z_* = 30 Å for all models in order to avoid the spurious influence of electric charges from one cell on another. This leads to a vacuum layer of around 20 Å between periodic replicas of the system. These values have been proven to be reliable in other similar studies [58]. The bulk cell parameter for silver has the value of 4.08 Å (experimental value). The theoretical value for the bulk cell parameter obtained by using the BH functional and plane-wave calculations was 4.10 Å [51]. Our tests on total energy versus lattice parameter lead to a value close to 4.095 Å.

We used a 3 × 3 Monkhorst–Pack grid for the integrals in the Brillouin zone for the transversal direction while the periodicity along the *Z*-axis was modeled with a single k-point. Our initial tests indicates that the employment of a 4 × 4 grid leads to a difference of 0.06 eV in the Kohn–Sham energy (i.e., about 0.3 meV/atom). As basis sets, we used a double-zeta polarized basis set for the Ag atoms and a triple-zeta polarized basis set for the molecule. The energy shift of the LCAO basis set employed in “Siesta” was 50 meV for all atoms. This value was chosen to be smaller than the standard “Siesta” value of about 200 meV, thereby allowing us to produce orbitals with larger cutoff radii in order to accurately simulate the long-range interactions. The grid used to calculate the integrals and to represent the charge density and potentials was defined by its plane-wave cutoff [52–53]. The value employed in our calculations was 250 Ry.

The systems were relaxed by keeping the three bottom layers of silver pinned to their positions, while all other atoms were allowed to relax to a maximum gradient of 0.02 eV/Å. Additional details on the geometric structures and relaxation procedure for molecule are presented in the next section.

The exchange–correlation (xc) functionals used in DFT are plagued by one- and many-electron self-interaction error (SIE) [59]. In the case of correlated electrons this effect may lead to significant errors. One of the most widely employed methods to correct SIE in xc classical-approximate functionals is the well-known DFT+U approach [60] (see Supporting Information File 1 for details). We applied the DFT+U corrections to the 3d orbital of the TM atom in all TMPP systems. For *J* we used the value of 0.2 eV (corresponding to a small hopping probability), while for *U* we employed the computed best-fit values as indicated in literature for metal phthalocyanine systems [61–62]. The complete list of values for *U* is given in Supporting Information File 1, Table S1. Since these values were tested only for a limited number of exchange–correlation functionals, we compared the density of states obtained using BH or the Perdew–Burke–Ernzerhoff (PBE) functional [63]. The data presented in Supporting Information File 1, Figure S1, shows that the DOS are almost identical for VPP, MnPP and FePP, while for three other molecules a qualitative agreement occurs. Since the main effect of DFT+U is to shift orbitals according to the *U* and *J* values, and the DOS for the two exchange–correlation functionals are similar it may be argued that the values reported in [61–62] can be also used for the BH functional.

## Results and Discussion

### Geometric properties

**Figure 1:**
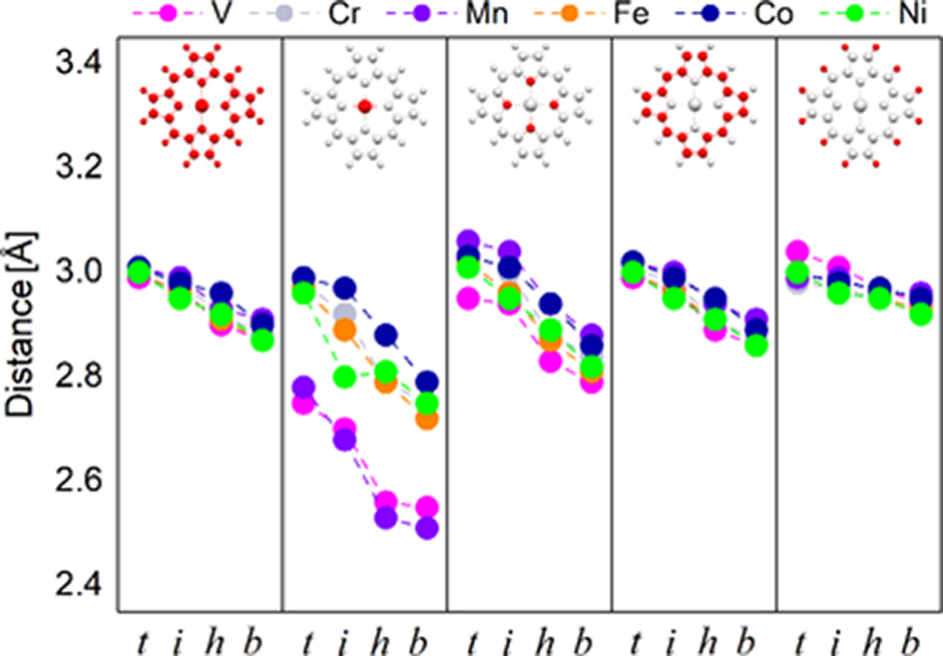
Graphic representation of the molecule–surface distances for all chemical species in TMPP. The images at the top indicate from left to right: the whole molecule, the transition-metal atom, N, C and H. The positions of the TM atom on top of Ag(111) are labeled as “*t*” (top), “*i*”(intermediate), “*h*”(hollow) and “*b*” (bridge).

In Figure 1 we summarize the molecule–surface distances. The positions of the central TM atom are labeled using the following symbols: “*t*” for top position, “*i*” is an intermediate position (no symmetry), “*h*” is the hollow position and “*b*” is the bridge position. The values represent the difference between the average *Z*-coordinate of the Ag atoms in the surface and that for the atoms in the molecule. These values were computed for different atom types as explained in the caption of Figure 1.

We note that the average distance between the molecule and the surface range from 2.82 to 3.00 Å with an almost linear decrease from the top position (largest values) to the bridge position. The differences between the five investigated molecules are negligible. A more detailed analysis shows that the same differences are present for the TM–surface values (second panel from left in Figure 1). Precisely, the V and Mn atoms have a clear trend to sit lower than the average values of all the other atoms. In addition, in the bridge position, they are about 0.2 Å lower than the average values of all other TM atoms. This hints to differences in the interaction mechanisms that are to be investigated later.

Next we investigate the nitrogen atoms. Here we see the largest slope in the position–energy curve among all chemical species in TMPP. Indeed, the molecule–surface distances vary over more than 0.2 Å from top to bridge position, proving that the nitrogen atoms play an important role in discriminating between different adsorption positions. In contrast, for carbon and hydrogen atoms this effect is minimal. Also for these two types of atoms the differences between the five TMPP molecules are minimal, which is no surprise, due to the geometric structure of the molecules. All TMPPs share the same organic moiety, i.e., the only difference between the molecules is the central metal atom. The interaction between TM atom and molecule takes place via the four nitrogen atoms, there is no direct influence of the TM atom on the carbon or hydrogen atoms. For VPP and MnPP we note the largest differences between the position of the metallic center and the plane of nitrogen atoms. Since the differences reach 0.3 Å we estimate the energetic penalty induced by the displacement of the TM atom along the axis perpendicular to the center of mass of the molecule. In Supporting Information File 1, Figure S4, we present results for the total energy as a function of the position of V and Mn atoms in free TMPP. For this simple model we get energetic penalties around 0.07 eV for MnPP and 0.18 eV for VPP, respectively. We note that these values are obtained for relaxed, isolated molecules by simply translating the TM atoms. Further relaxation of the positions of nitrogen atoms will probably diminish these values, which should be considered as the maximum possible values.

It is interesting to observe that the dependence on the adsorption position is weaker from left to right in the panels of Figure 1, suggesting that the interaction between TM atom and silver surface depends locally on the adsorption symmetry. For all the other atoms the dependence is probably indirect and is induced by the proximity of the TM atom, since the dependence between position and distance is decreasing with the distance from the TM atom (larger for nitrogen, intermediate for carbon and minimal for hydrogen).

**Figure 2:**
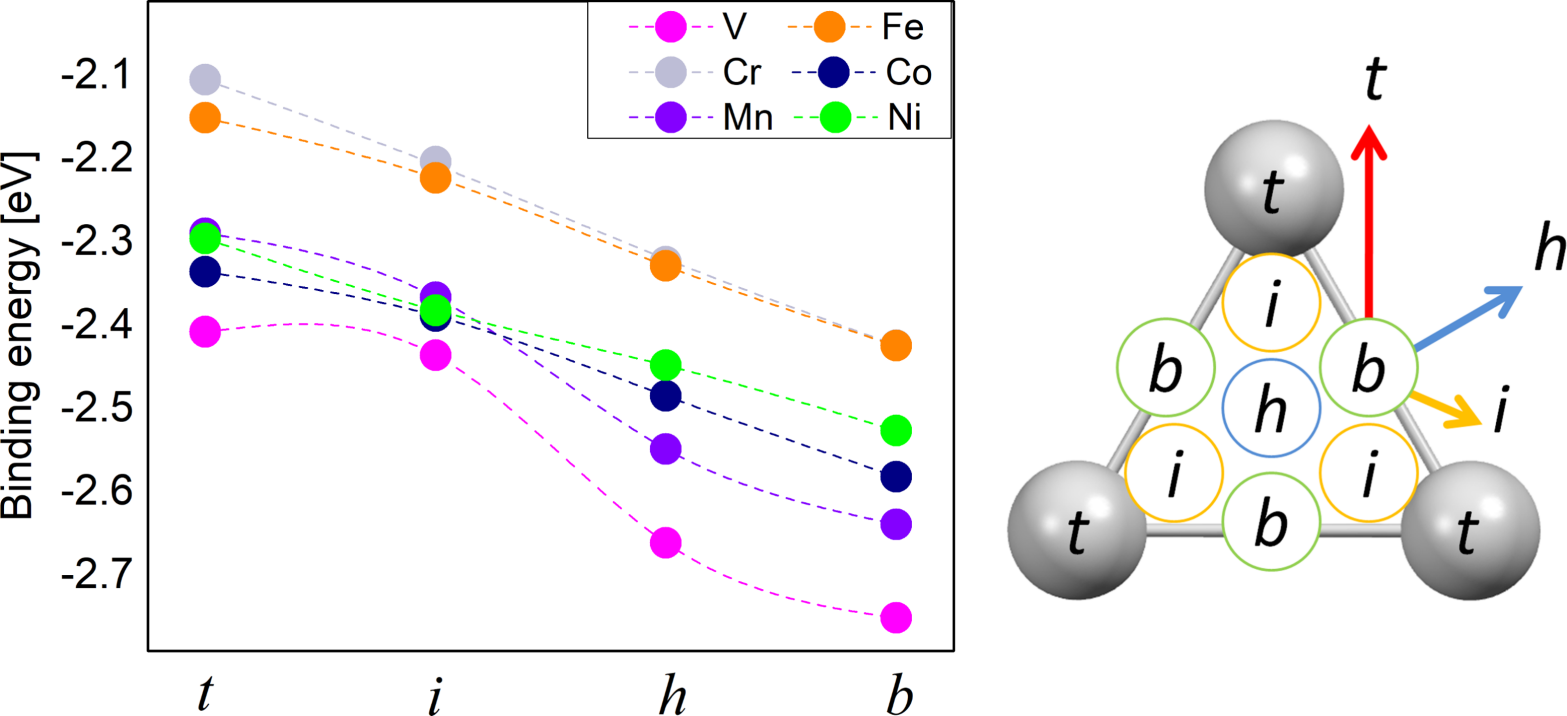
Left: binding energy values, 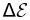, for all systems and relative molecule–surface positions. Right: symmetry-equivalent positions on the Ag(111) surface, according to our notations. The color arrows indicate qualitatively the jumping probabilities over the energy barriers in the systems (blue = highest, red = lowest).

The results for the binding energies of TMPP and silver are summarized in Figure 2. Due to symmetry considerations, the four points investigated by us will cover most of the unit cell of the Ag(111) surface (see the right panel of Figure 2). There is an almost linear dependence between energy and position, with two exceptions: VPP and MnPP. In these two cases the intermediate position shows a shift from the linear trend, most probably as a result of the low symmetry of the “*i*” position. In addition, this may also be connected to the position of the TM atom with respect to the surface as shown in Figure 1. We note that the values close to 2.5 eV are similar to those reported in other studies on similar systems [26]. The van der Waals-corrected binding energy of CoTPP to Ag is 274 kJ/mol (ca. 2.84 eV) [26]. Remarkably, in the same study the PBE binding energy is 54 kJ/mol (0.56 eV), which means a decrease by a factor larger than four. This points out the importance of the van der Waals component in the interaction energy and also gives a hint that the chemical bond between TM and silver is probably a weak bond, since the value of 0.5 eV is the qualitative limit between physically and chemically bonded systems [64]. For VPP and MnPP the interaction between TM atom and substrate is sufficiently strong to overcome the energetic penalty caused by the deformation of the planar structure of the TMPP molecule (see the comment in the paragraph above). Indeed, the two systems display the strongest binding energies among all systems.

For all systems we found that the bridge configuration is the most stable. However, the maximum difference between the total energies of different configurations is around 0.25 eV, which is a relatively small values, close to the accuracy of DFT. The ordering of the binding energies is (from highest to lowest): VPP, MnPP, CoPP, NiPP, FePP and CrPP (the last two have identical binding energies). These close values may explain the complex geometries that were found in the experimental studies. For example, studies on systems similar to CoPP (i.e., CoTPP) have shown that the real Co positions might deviate from the exact hollow sites and rather display a quasi-fcc or/and quasi-hcp registry with the underlying Ag(111) lattice [65].

By analyzing the binding energy as a function of the position we get a qualitative picture of the dynamics of the adsorbate on the surface as well as hints on the self-assembly and formation of covalently bonded structures. Differences in binding energies are an indicator of how easy the molecules change their positions on the surface in order to produce self-assembled structures at room temperature. An energy barrier around 0.03 eV would allow for free migration of the molecules on the surface at room temperature. We see that for all TMPP–Ag(111) systems the smallest barrier between two points is slightly smaller than 0.1 eV. This is the energy difference between the bridge and hollow positions. This indicates that the majority of the molecules are pinned to stable positions at room temperature, according to a Boltzmann distribution of populations. Nevertheless, lateral interactions as well as a temperature increase may easily provide enough energy to overcome a barrier of 0.1 eV. According to the data in Figure 2 the self-assembly will take place by successive jumps between bridge and hollow positions of the TM atom. Two comments should be made about this model: first, our previous discussion refers to the most probable scenario. Due to the large prefactor in the Boltzmann distribution law, even at a sub-monolayer coverage a large number of molecules are mobile on the surface. Also, following the same argumentation we can say that the direction of migration is not restricted from bridge to hollow positions. While the energy barrier along the bridge and hollow positions is minimal, a number of molecules will also move from bridge to the intermediate or even the top position (see the arrows in Figure 2). The probability of such trajectories is significantly lower, due to higher energy barriers. The second comment is that a full description of the energy barrier should include the rotational degrees of freedom. In our model the molecule performs translations between the points indicated at the right side of Figure 2. A further minimization of the barrier may occur if the transition between two equilibrium points is made by combining translation and rotation of the molecule. Since this is beyond the scope of our paper, we only draw attention that the energy barriers in Figure 2 are valid for the model of a molecule that is performing translations on the surface. In other words, these are maximal values, because in the presence of additional rotational degrees of freedom the energy barrier may diminish.

Moreover, we see a relatively constant energy difference between the four positions investigated by us. This is an important information for the process of formation of a covalently bonded network at surfaces where a mismatch between substrate and TMPP (or a derivative) is expected to occur. In such cases, TMPP may be forced to assume all four positions presented in Figure 2. The interplay between specific geometric parameters of the molecular networks and the energy barrier that can be overcome by the assembly forces is essential for understanding the whole process. Since the energy depends almost linearly on the position for TM = (Cr, Fe, Co and Ni) it is reasonable to assume that in these cases the process will follow similar dynamics. For VPP, and partially for MnPP, a larger gap occurs between the “*i*” and “*h*” position, suggesting that the formation of covalently bonded structures will be different compared to that of CrPP, FePP, CoPP and NiPP.

**Figure 3:**
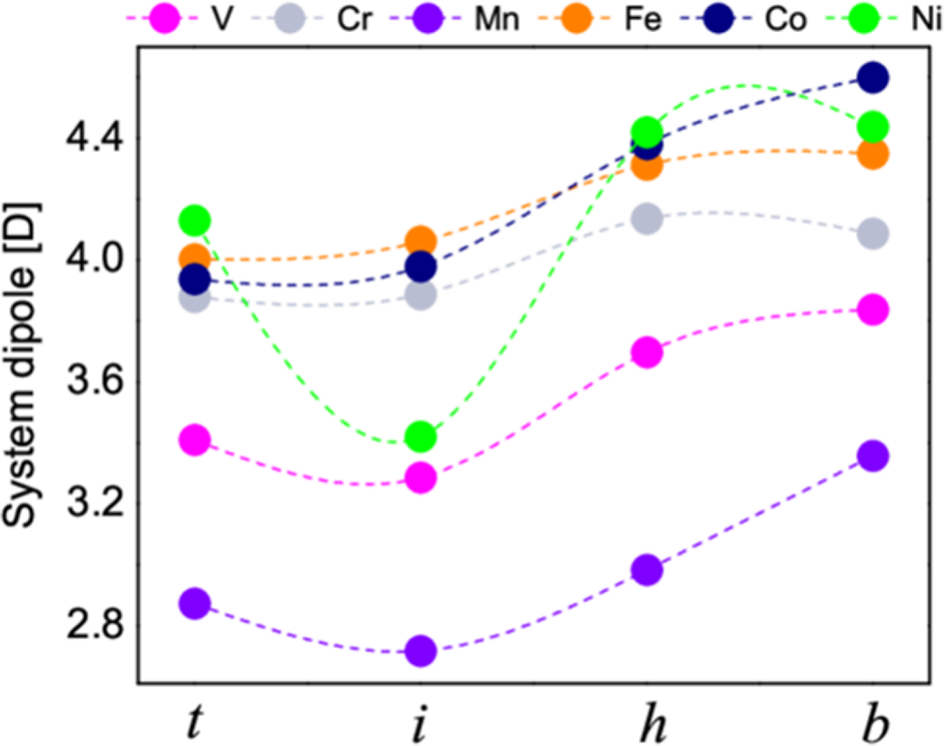
Dipolar moments for all TMPP–metal-surface systems and all relative positions between molecule and surface.

In Figure 3 we present the dipolar moments of the molecule–surface systems. The values are calculated as integrals over the entire unit cell 
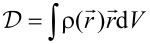
[52]. Because of symmetry considerations, the dipolar moments are perpendicular to the surface and oriented from the surface to the molecule. The values in Figure 3 are the components along the *OZ*-axis of the dipoles. We observe a trend that is qualitatively similar to that of the binding energies. The smallest values are obtained for the top positions while the largest ones are obtained for the bridge positions. Most probably this is the result of decreasing the distance between molecule and surface. Since the molecule is closer to the surface, the molecule–surface charge transfer becomes more important, leading to higher dipolar moments. The values for the dipolar moment are relatively large suggesting a noticeable charge transfer between molecule and surface.

The dipolar moments of the VPP and MnPP are the smallest among all systems. This further strengthens our hypotheses that the two systems have an interaction mechanism to the surface that is different from the rest. Secondly, for NiPP we note an anomalous behavior at the intermediate point. This is correlated to the anomalous magnetic behavior, to be discussed in the section dedicated to the electronic structure and magnetic properties.

Let us comment on the practical importance of the results in Figure 3. Since the dipolar moments of the molecule–surface systems are all parallel and perpendicular to the surface, this leads to a repulsion between the molecules. It is important to estimate whether this repulsion energy is comparable to the energy differences between the points “*t*”, “*i*” “*h*” and “*b*”. Indeed, we expect the self-assembly process to be driven by the opposing trends of assuming the energetically most favorable configuration and intermolecular repulsion. In addition, migration on the surface occurs through passing from one of the four positions investigated by us (or close to those) to another. We thus estimate the repulsion energy of the dipoles by taking into account the size of the molecule (about 10 Å) and the value of 4.5 Debye (maximum value in Figure 3). The result is close to *kT*/2. Hence, it could play a role in the molecule dynamics on the surface at room temperature, because since the value of *kT* at room temperature is close to 0.03 eV. The dependence of the dipolar interaction energy on 1/*r*^3^ will dramatically decrease if linkers are used in the TMPP structure (for example TPP). In short, while the dipolar repulsion will not block migration from a position to another, it will play an important role in the dynamics of the adsorbate on the surface.

### Electronic structure and magnetic properties

We start our discussion with the magnetic properties of the adsorbed molecules. We found that the magnetic moments are stable with respect to a change in the position of the TM atom. Indeed, with the only exception of NiPP, we have similar magnetic moments for all four adsorption positions. The values are listed in Table 1.

**Table 1:** Magnetic moments, expressed in μ_B_, for all TMPP systems in vacuum and all adsorption positions.

molecule	vacuum	*t*	*i*	*h*	*b*

VPP	3.00	2.97	2.92	2.88	2.85
CrPP	4.00	3.69	3.69	3.62	3.57
MnPP	5.00	4.73	4.74	4.73	4.72
FePP	4.00	1.79	1.80	1.75	1.69
CoPP	1.00	0.80	0.82	0.81	0.79
NiPP	0.00	0.01	1.77	0.00	0.01

Our results are similar to those obtained for porphyrin-based molecular nanowires [66]: The total magnetic moments of TM-PNWs are calculated to be approximately 4.0, 4.8, 1.0 and 0μ_B_ per unit cell for TM = Cr, Mn, Co and Ni, respectively. In order to comment on the dependence between the position and magnetization we remind that the magnetic moments are the result of the crystal-field splitting of the d-orbitals of the TM. Hence, they depend critically on the geometric properties. For example, the magnetic properties of porphyrin on porous graphene-like carbon nitride gives values such 3μ_B_ for Fe, 0.91μ_B_ for Co and 1.49μ_B_ for Ni [67].

The magnetic moments obtained for the adsorbed molecules are close to those of the free molecules, with a single exception. For FePP we obtain a magnetic moment of 4μ_B_ in the isolated molecule, while the adsorbed molecule has a magnetic moment of 1.69μ_B_.

The magnetic moments for different TMPP molecules are separated by approximately 1μ_B_ as a consequence of the gradual filling of the 3d orbitals. The largest magnetic moment is obtained for MnPP. The result is close to the result predicted by Hund’s rules for a single atom (5μ_B_). The smallest value of zero was obtained for NiPP. In this case, there is only a change of the magnetic moment as a result of changing the adsorption site.

At the low-symmetry “*i*” points, the effect on the electronic structure of the molecule is strong enough to change the magnetization of the adsorbed molecule (see Supporting Information File 1, Figure S5 left panel, for details of the projected density of states of NiPP at the “*i*” and “*b*” points). The density of states of the “*i*” and “*b*” positions shows that in the bridge position the 3d orbital at 1.3 eV above the Fermi level is relatively localized from an energetically point of view. At the “*i*” position it interacts with the silver substrate, leading to a very broad density of states that is in the vicinity of the Fermi level. Consequently, the spin-down states are occupied and the system has a non-zero magnetic moment. This effect is reversed for the deep orbital at −3.7 eV, which is localized (i.e., no coupling to energy bands of Ag) in the “*i*” states, while there is a clear interaction with the metallic substrate at the bridge position. All this will lead to a non-zero magnetic moment in the “*i*” state. As a result, the dipolar moment is also influenced, as discussed above. If we extrapolate the values for top and hollow positions, a large magnetic moment is correlated with a decrease of the dipolar moment by more than 1 D. In order to see if this is a computational artifact, we have repeated the calculations by using a second value for *U* in the DFT+U method. This way we get a magnetic moment of 1.65μ_B_ for *U* = 4 eV, compared to the value of 1.77μ_B_ obtained for *U* = 6 in the intermediate position. In both cases the magnetic moment remains zero at the bridge position.

**Figure 4:**
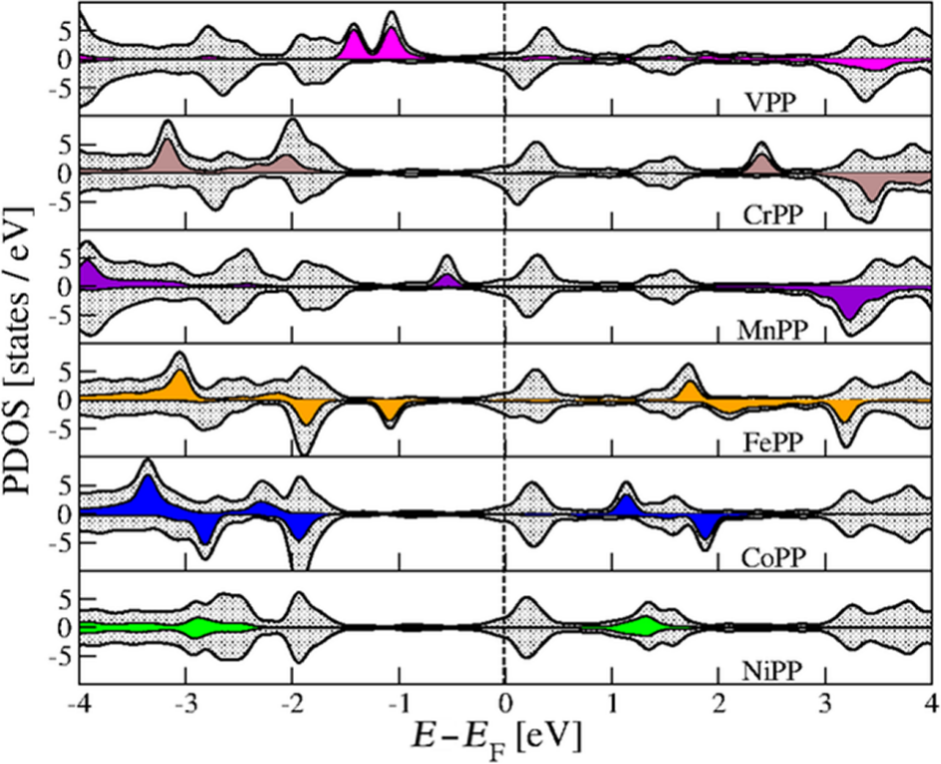
Density of states projected over the molecule (gray) and the central TM atom in the molecule. Spin-up and spin-down are represented by their corresponding orientations. Fermi level of the metallic substrate was set to zero.

In order to rationalize the values for magnetic properties above, we further investigated the electronic structure of the adsorbed molecules. The density of states projected over the atoms of the adsorbed molecule are given in Figure 4, together with the PDOS of the transition metal in each structure. For each system we performed a qualitative analysis of the contributions to the frontier orbitals in the adsorbed molecule by integrating the PDOS of all atoms in a range of 0.1 eV around the corresponding energies. The results are presented in Supporting Information File 1, Table S4.

We see from Figure 4 that all adsorbed TMPP molecules have a LUMO orbital close to 0.2–0.3 eV above the Fermi level of Ag(111) surface. The major contributions to this orbital are from the 2p*_z_* orbitals of the nitrogen atoms. Small contributions of the TM atom are present only for VPP and FePP. There are also contributions for NiPP, but only in the “*i*” position (not shown in the Figure 4). For VPP we also found a small contribution of the 4s orbital of vanadium to the HOMO orbital (about 5%). The HOMO results are more disperse. While for MnPP we have a HOMO with an important contribution of the d-orbitals at −0.5 eV, for NiPP the HOMO has zero contribution from d-orbitals as it is located −1.9 eV below the Fermi level. As expected, there are similarities between the electronic structure of VPP and MnPP. Essentially the differences between the two PDOS are given by the peaks of the d-orbitals (−0.5 eV for MnPP and −1.1 eV for VPP). The contributions to HOMO (and HOMO−1) are also relatively disperse and spin-dependent, as indicated in Supporting Information File 1, Table S4. The 3d orbitals of metal and the carbon atom orbitals play an important role here. For MnPP we found small contributions of the 2s orbitals of nitrogen. The HOMO–LUMO gaps are in agreement with results in literature [68]. They range from 2.0 eV (CoPP, NiPP) over 1.0 eV (FePP) to 0.5 eV (Mn).

A semi-quantitative investigation of the charge migration can be done by using the quantity


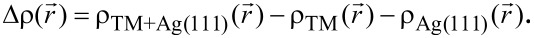


Molecule–surface charge transfer occurs through a whole range of effects, from chemical bonds to Pauli push-back of electrons from the close shells and from the surface [69–71]. The accumulation of charge in the region between the molecule and the surface indicates the formation of a chemical bond while a depletion is connected to molecule–surface repulsion. The presence of nodal planes between molecule and surface is an indication for the lack of chemical bonds.

**Figure 5:**
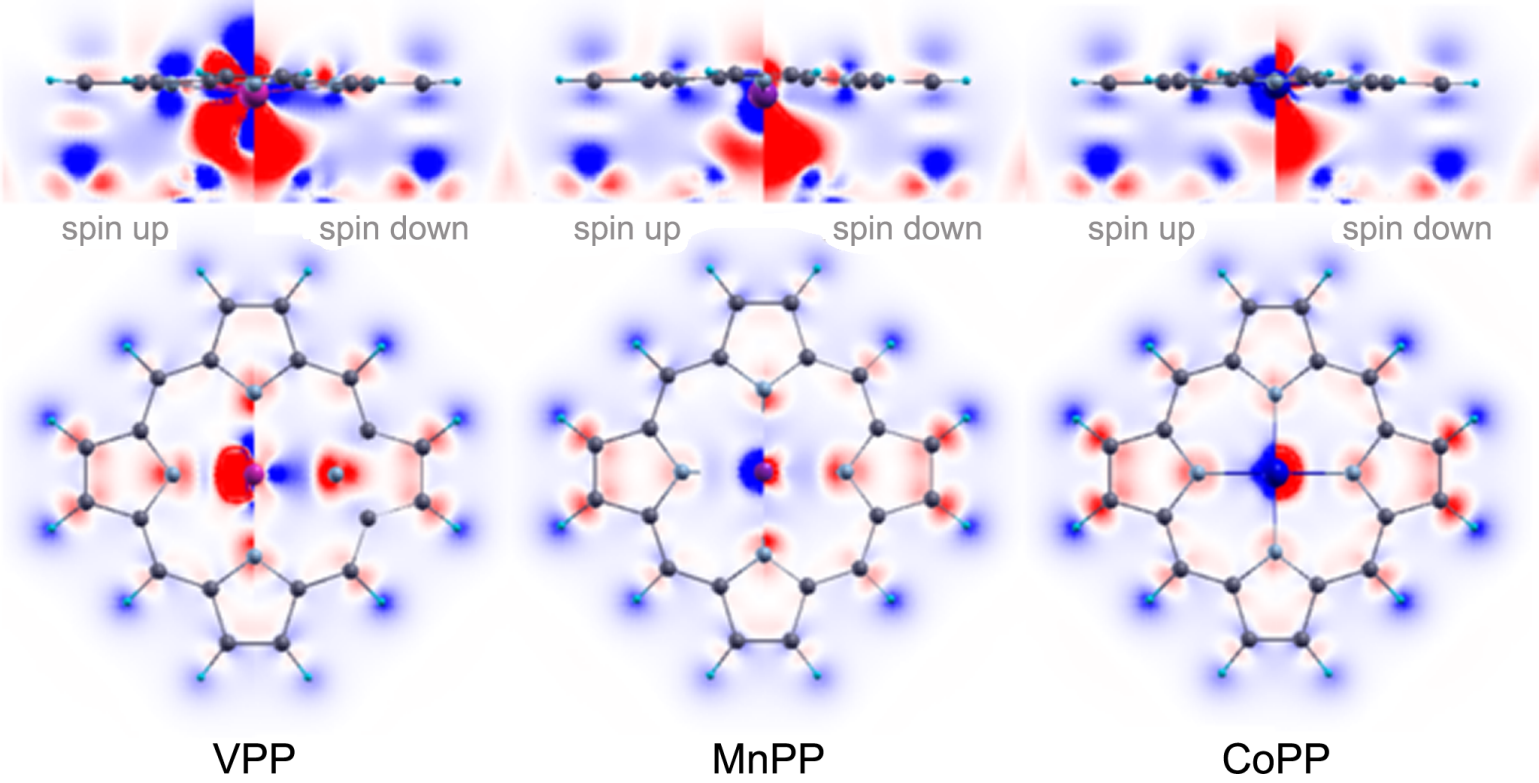
The values of 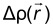 for selected TMPP systems (M = V, Mn, Co; red: positive values, blue: negative values). Min/max values ±0.0015 *e*/bohr^3^. We use two perpendicular planes to plot the figures, i.e., the plane including the atoms of TMPP (bottom) and the plane perpendicular to it, including the TM atom (top). Details for spin-up and spin-down are presented in each half of the three images.

The values of 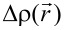 for selected TMPP systems (TM = V, Mn, Co) are summarized in Figure 5. In the case of VPP there is an increase in both spin components of 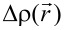 in the region between the vanadium atom and the silver surface. For MnPP and CoPP, only the spin-down component exhibits bond-like behavior. For the spin-up component a nodal plane for 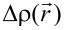 is present between TM atom and silver surface. As discussed above, the accumulation of electrons between two atoms is an indication of the chemical interaction while a nodal plane may correspond to an anti-bonding configuration. We note the presence of a stronger chemical bond for vanadium, where both spin components participate to the bond. This is consistent with the fact that, in general, TMPP molecules form a relatively weak chemical bond to the surface [26]. The exception here is VPP (Figure 5, top left panel). This corroborates the results of binding energy presented in Figure 2 in which VPP exhibits the largest binding energy of all systems. At a qualitative level in Figure 5 it can be seen that the bond in MnPP (i.e., the continuous red area between TM atom and surface) is slightly stronger as in CoPP, as shown in Figure 2.

In the organic moiety of the molecule the hydrogens act as electronic donors with a decrease of the population over all atoms. The charge is transferred partially to the carbon atoms. An interesting fact is that this transfer does not occur symmetrically, for all hydrogen–carbon bonds. Instead, we see strong increase of the charge on the carbon atoms in the pyrrole for two opposite rings, while for the other two the effect is significantly weaker. Most probably this is the effect of the surface, since the data in Figure 5 are computed for the bridge orientation (i.e., the most stable from the energetic point of view).

Let us investigate the Voronoi populations for the transition metals in free and/or adsorbed molecules. The results in Table 2 show that the adsorption leads to an important decrease of the electronic population on the TM atom, i.e., a positive charge is accumulated on the TM atom. This loss of electronic population is in agreement with the results in Figure 5, where it can be seen that electrons are transferred to the molecule–surface bond. Also, the reduction of the oxidation state of Co and Fe in metalloporphyrins adsorbed on Ag(111) was shown experimentally [26].

The total Voronoi charges of the adsorbed molecules were calculated by summing up charges over all atoms. The last column in Table 2 show that these values are close to −0.2*e*, with a maximum for VPP and a minimum for CrPP. The large values for VPP and MnPP are connected to large values of the binding energy. For the remainder of the molecules there is no correlation between the values of total charge of the adsorbed molecule and the binding energy. This clearly shows the presence of a different interaction mechanism in VPP and MnPP. The total Voronoi charges of the adsorbed molecules in all positions are reported in Supporting Information File 1, Table S3 and shows fluctuations up to 50% percent between the equilibrium (“*b*”) and the point with the highest energy (“*t*”).

Perhaps the most interesting result of the Voronoi population analysis is the lack of correlation between the total charges of the molecules and the dipolar moment. Indeed, no clear correlation can be found between the data in Table 2 and the values of dipolar moment in Figure 3. Figure 5 shows that this can be explained by the failure of the concept of “atomic population”, as computed by various methods. While the Voronoi population is assigned to specific atoms, it can be seen in Figure 5 that the charge reorganization upon adsorption is important for the entire volume between molecule and surface. Consequently, the dipolar moments computed by integration over the unit cell are only partially correlated to the atomic populations.

**Table 2:** Voronoi populations, expressed in |*e*| for the central TM atom for all TMPP molecules. From left to right: single molecule at relaxed geometry; single molecule at the adsorption geometry; molecule adsorbed at the bridge site and total population over the entire adsorbed molecule.

molecule	relaxed	distorted	adsorbed/TM	adsorbed/total

VPP	0.310	0.237	0.133	−0.276
CrPP	0.542	0.546	0.508	−0.150
MnPP	0.339	0.362	0.209	−0.190
FePP	0.302	0.262	0.125	−0.187
CoPP	−0.107	−0.056	−0.147	−0.167
NiPP	0.148	0.119	0.111	−0.164

We monitor the effect of molecular deformation upon the electronic population (second column in the Table 2) in order to separate the chemical influence of the surface from the physical effect, i.e., deformation. Molecular deformation is an important factor influencing the electronic population of the transition metals, and it can be held responsible for 30–50% of the population change in the adsorbed molecule. The difference is probably pushed in the molecule–surface bond, as shown in Figure 5.

### Molecule–surface interaction

It was shown that upon the adsorption of (Fe,Co)(II)-tetraphenylporphyrin on Ag(111) a covalent bond is formed [26]. The bond is formed between the metallic center of the porphyrin derivative and the silver surface. For Zn(II)-tetraphenylporphyrin no bond formation was observed, indicating that the bond strength depends on the electronic structure of the metal center. The same study indicates that the interaction of the coordinated metal center with the Ag substrate can be significantly reduced due to the coordination of NO group to the metal center. It was concluded that the TM–Ag bond can be described locally as a covalent two-orbital/two-electron bond between the Ag 5s and TM 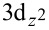 orbitals [26].

Our analysis of the PDOS for the 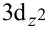 orbitals for FePP and CoPP indicates the presence of two peaks around Fermi level, which correspond to the bonding and anti-bonding mechanism (see Supporting Information File 1, Figure S5, right). The analysis of the 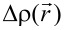 quantity displays shapes specific to a covalent bond, i.e., charge accumulation between the atoms in direct interaction. For one of the two spin components of charge density charge accumulation is present, while there is a nodal plane in the 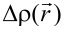 plot for the second component of the spin. The exception is the VPP molecule where the chemical nature of the interaction is clear for both spin components. A dipolar interaction between adsorbed molecules is also likely to appear due to relatively large values of the dipole moments. These dipolar moments are a consequence of the charge transfer between molecule and surface. The Voronoi population analysis indicates a charge of around −0.2|*e*| in the adsorbed state over the entire TMPP molecules.

In order to estimate the role of van der Waals interaction we remind that for other aromatic systems the binding energy obtained without van der Waals corrections, e.g., using only the PBE functional [26]) are a factor of four smaller compared to energies including van der Waals correction [26,72]. By taking into account the van der Waals interaction, the binding energy dramatically increases due to the interaction between π-system and metallic surface. For porphyrin adsorbed on Ag(111) binding energies of around 2.5 eV were reported [58]. These values are close to those reported in Figure 2. By comparing our data with those available in literature, it can be argued that van der Waals interaction is the dominant component in the binding energies reported in Figure 2, while a weak chemical bond is responsible for the direct metal–surface interaction (see Figure 5).

The interplay between charge transfer and magnetic properties can lead to a range of effects from Kondo scattering, mixed valence bonding or quenching of magnetic moments [73–77]. Since the quantitative estimation of the Kondo effect is beyond the limits of mean-field theories (such as DFT) we can provide a qualitative discussion based on experimental results for the Kondo effect in similar systems. It was shown that Kondo effect of organo-metallic compounds adsorbed on coinage metals is sensitive to the manipulation of the chemical bonds [73–74]. The Kondo effect in Co-porphyrins on Au(111) can be switched on or off by binding a NO group to the molecule [73]. Also, the changes in molecular conformation can be used to achieve similar effects [74–75]. It was shown that chemical bonds formed between substrate and molecule may lead to a deformation of the planar structure of the Co-porphyrine or Co-phthalocyanine resulting in the increase of the molecule–cobalt distance up to 0.8 Å [75]. Our data also indicate relatively important fluctuations of the metal–surface distance as well as of the dipolar momentum, depending on the position of the TM atom relative to the silver atoms. This position can be tuned by “forcing” the atom to sit on different positions. A simple strategy to do this is to use lateral linkers for porphyrin, such as phenyl or biphenyl groups. These linkers will tend to maximize their van der Waals interaction with the surface, resulting in change of the position of the TM atom relative to the silver atoms. Nevertheless, the use of linkers will not influence significantly the local environment of the TM atom. This may result in a mechanism for the manipulation of the Kondo physics in the molecule, inspired by those commented above [73–75], since the changes in the dipolar effect and in the TM–surface distances may be sufficient to change the interaction mechanism between the conduction electrons in the surface with a possible Kondo screening.

A second comment is related to the crystal-field splitting around the TM centers, which may lead to a quenching of the calculated magnetic moments. The example of NiPP is already present and discussed in this paper. The completely non-symmetric point “*i*” allows for the presence of a total magnetic moment. This is correlated with an increase of the dipolar moment, caused by the lowering of the position of Ni in NiTPP with respect to the surface. By going to any of the highest symmetry points around it, the magnetic moment collapses. The DFT results can fail to accurately reproduce all these situations mainly because of the underestimation of correlation effects in DFT. Consequently, spin effects are described with limited accuracy for both TM and ligand spin. The latter is just barely present in the calculations of adsorbates [77]. For example, the spatial representation of the spin density (Supporting Information File 1, Figure S6) shows that the spin is concentrated almost exclusively at the TM atoms, with a negligible (less than 1%) ferimagnetic contribution of the four nitrogen atoms surrounding the TM atom. It was shown that at the Co-phtalocyanine/Au interface, the magnetic moment of the Co atom is completely quenched by the molecule–substrate interaction [76]. As commented in the paragraph on the Kondo effect, the presence of additional linkers may lead to a perturbations in the crystal field that ultimately could lead to a quenching of the magnetic moments reported on Table 1 due to symmetry and TM–surface interaction strength, which may lead to a change in the metal–surface distance. Ultimately, this may provide a simple mechanism of controlling the spin at the interface.

We conclude that the TMPP–Ag(111) interaction is a complex mechanism that involves a weak chemical bond between metal and surface, a relatively important charge transfer and an important van der Waals interaction. Molecule–surface charge transfer is reflected in the varying electronic population of the central TM atom, which in turn is decisive for the magnetic moment of the adsorbate molecule.

## Conclusion

We have presented a first-principles analysis of the physical properties of six transition-metal porphyrines adsorbed on a Ag(111) surface. Our DFT calculations are based on the exchange–correlation functionals developed for the study of van der Waals interactions. These are combined with the DFT+U method in order to take into account the correlation effect in the 3d orbitals of the transition metals.

Four positions of the metallic atoms on top of Ag(111) were investigated for each molecule in order to understand the influence of molecule translation on electronic structure and properties. Based on the values of binding energy and molecule–surface geometry we have concluded that the bridge position is the most favorable for all transition-metal atoms in our investigation. The total binding energies range between 2.4 and 2.7 eV at the bridge position, while at the top position the values are between 2.1 and 2.4 eV. The energy difference between the latter one and the hollow position is around 0.25 eV, while the energy difference between bridge and hollow is less than 0.1 eV.

The analysis of physical properties of the adsorbed molecule indicates that the binding mechanism is complex, involving a small metal-dependent chemical interaction, a relatively large dipolar interaction due to the molecule–surface charge transfer and van der Waals contributions.

The magnetic moments are relatively stable with respect to translational degrees of freedom of the molecule. The only system where we found a position-dependent magnetic moment is NiPP, where in the low-symmetry point a magnetic moment close to 2μ_B_ is obtained, while for other adsorption sites the magnetic moment is zero.

Detailed information collected for a range of adsorption sites may find utility in the understanding of the surface dynamics as well as in the design of experiments oriented to the fabrication of nanostructures with controlled functionalities. Also, the comparison between properties of the six transition-metal porphyrines on silver may serve in the future as a guiding tool for the design of such functionalities.

## Supporting Information

File 1 Additional computational data.
