# Supplementary material for: The effect of translation on the binding energy for transition-metal porphyrines adsorbed on Ag(111) surface
Source: Beilstein J Nanotechnol. 2019 Mar 13;10:706–17. doi: 10.3762/bjnano.10.70 (PMC6423576; doi:10.3762/bjnano.10.70)
Supplement: File 1 — Additional computational data. [file Beilstein_J_Nanotechnol-10-706-s001.pdf]

BEILSTEIN JOURNAL OF NANOTECHNOLOGY

Supporting Information

for

The effect of translation on the binding energy for transition-metal porphyrines adsorbed on Ag(111) surface

Luiza Buimaga-Iarinca and Cristian Morari

Beilstein J. Nanotechnol. **2019,** *10,* 706–717. doi:10.3762/bjnano.10.70

Additional computational data

License and Terms: This is a supporting information file under the terms of the Creative Commons Attribution License (http://creativecommons.org/licenses/by/4.0). Please note that the reuse, redistribution and reproduction in particular requires that the authors and source are credited.
The license is subject to the *Beilstein Journal of Nanotechnology* terms and conditions: (https://www.beilstein-journals.org/bjnano)

Computational details

Binding energy

In order to compute the BSSE-corrected binding energy we first calculate the quantity:

$$\Delta E_1 = E^{MS}_{MS} - E^{MS}_{M} - E^{MS}_{S} \tag{S1}$$

where M denotes the molecule, S the surface. Superscript index is used to label the geometric configuration of the system in interaction, while the subscript index represent the parts of the system (molecule, surface or complete system) where the atoms are replaced by corresponding 'ghost atoms' (see reference [1] for further details.)

Eq. S1 represents the molecule-surface binding energy if the geometry of the adsorbed molecule suffer little deformation in the adsorption. Since the geometric structure of adsorbed molecule has a different geometry compared to geometric structure of the molecule in vacuum we add to the total binding energy of the molecule-surface system, the value

$$\Delta E_2 = E_A - E_V \tag{S2}$$

where E_V and E_A are the total energies of the molecule in vacuum (i.e. relaxed structure) and adsorbed (i.e. the geometric structure obtained in the adsorbed state). By combining the energies resulting from the Equations S1 and S2, we get the best estimation for the binding energy computed in the LCAO framework.

LDA+U corrections

The electronic correlation in the localized d orbitals of central atom of MPC is calculated using DFT+U corrections as implemented in SIESTA [2-4]. DFT+U Hamiltonian has the form:

$$\hat{H} = \frac{\bar{U}}{2} \sum_{m,m',\sigma} \hat{n}_{m,\sigma}\hat{n}_{m',-\sigma} + \frac{\bar{U}-\bar{J}}{2} \sum_{m \neq m',\sigma} \hat{n}_{m,\sigma}\hat{n}_{m',\sigma} \tag{S3}$$

where the summation runs over the projections of the orbital momentum (m and m') while $\bar{U}$ and $\bar{J}$ are the spherically averaged matrix elements for the screened on site Coulomb and hopping interactions respectively. In terms of Kohn-Shamm eigenvalues ε_j, this produces the total energy:

$$E_{DFT+U} = E_{DFT}[\{\varepsilon_j\}] + \frac{\bar{U}-\bar{J}}{2} \sum_{l,j,\sigma} \rho^{\sigma}_{l,j} \rho^{\sigma}_{jl} \tag{S4}$$

where $\rho^{\sigma}_{l,j}$ is the density matrix of the manifold taken into account.

Table S1: The values of U used in calculations. For all systems J was set to 0.2 eV.

System	VPP	CrPP	MnPP	FePP	CoPP	NiPP
$U[eV]$	4.1	4.1	4.0	4.4	4.0	6.0

Geometric properties

Electronic structure and charge transfer

Table S2: Magnetic moments, expressed in μ_B for NiTPP/Ag(111), for "i" and "b" position, for $U = 4$ and $U = 6$, respectivelly.

Position	i	b
$U[eV]$ =6	1.77	0.01
$U[eV]$ =4	1.65	0.02

Table S3: Voronoi total charges of the adsorbed molecules, expressed in $|e|$ for all TMPP systems and all adsorption positions.

Model	t	i	h	b
VPP	-0.203	-0.204	-0.255	-0.276
CrPP	-0.052	-0.071	-0.170	-0.150
MnPP	-0.123	-0.134	-0.170	-0.190
FePP	-0.091	-0.115	-0.150	-0.187
CoPP	-0.101	-0.117	-0.126	-0.167
NiPP	-0.076	-0.163	-0.127	-0.164

References

1. Boys, S.F.; Bernardi, F. The calculation of Small Molecular Interactions by the Differences of Separate Total Energies. Some Procedures With Reduced Errors. *Mol. Phys.* **1970**, 19, 553 - 566.

2. Ordejón, P.; Artacho, E.; Soler, J. M. Self-Consistent Order-N Density-Functional Calculations for Very Large Systems. *Phys. Rev. B* **1996**, 53, R10441 - R10444.

3. Soler, J. M.; Artacho, E.; Gale, J. D.; García, A.; Junquera, J.; Ordejón, P.; Sánchez-Portal, D. The SIESTA Method for Ab Initio Order-N Materials Simulation. *J. Phys.: Condens. Matter.* **2002**, 14, 2745 - 2779.

4. Dudarev, S. L.; Botton, G. A.; Savrasov, S. Y.; Humphreys, C. J.; Sutton, A. P. Electron-Energy-Loss Spectra and the Structural Stability of Nickel Oxide: An LSDA+U Study. *Phys. Rev. B* **1998**, 57, 1505 - 1509.

5. Perdew, J. P.; Burke, K.; Ernzerhof, M. Generalized gradient approximation made simple. *Phys. Rev. Lett.* **1996**, 77, 3865.

6. Berland K.; Hyldgaard P. Exchange Functional that Tests the Robustness of the Plasmon Description of the van der Waals Density Functional. *Phys. Rev. B.* **2014**, 89, 035412.

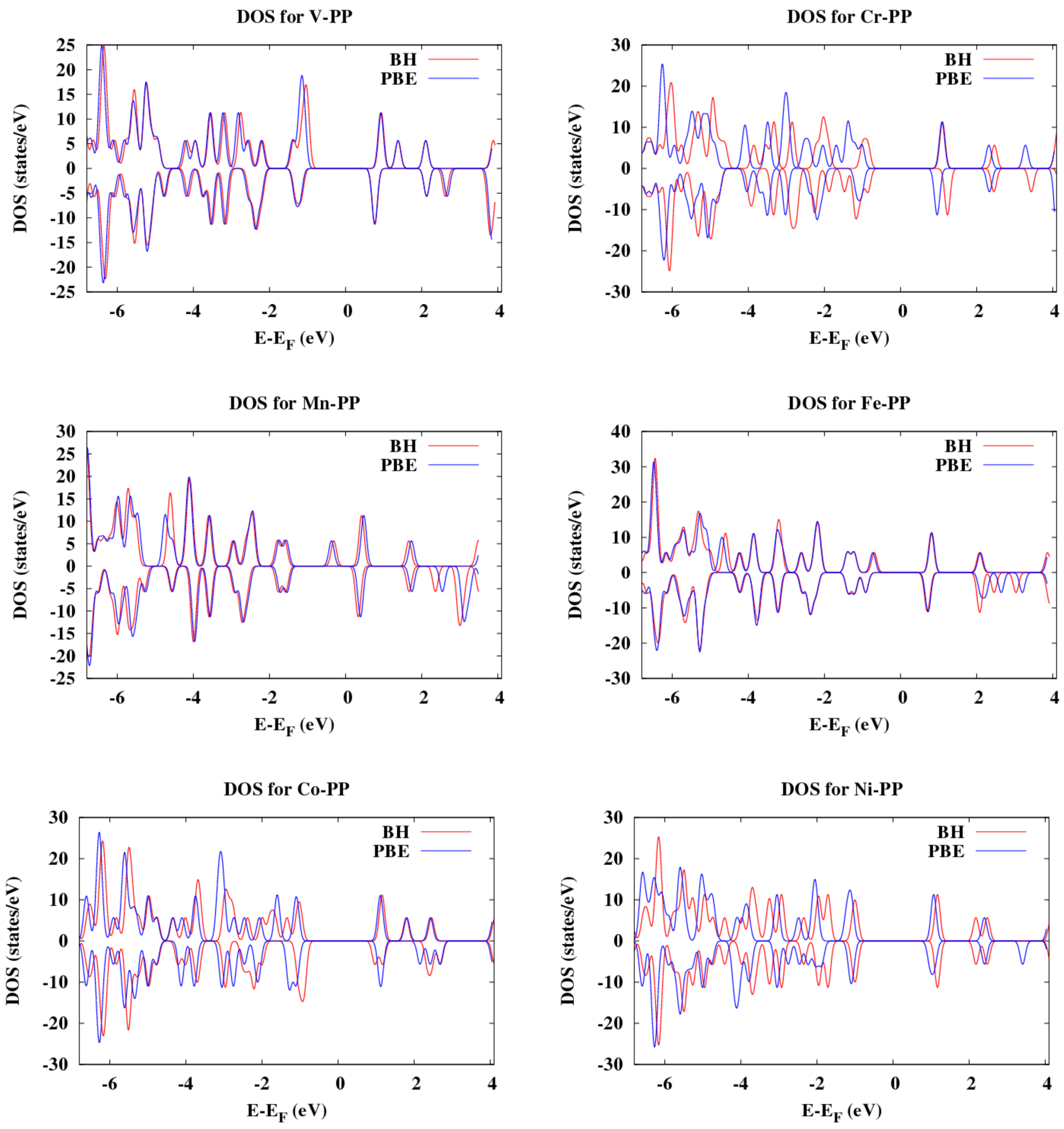

Figure S1: Density of states for the TMPP molecules for two types of exchange correlation functionals: PBE [5] and BH [6], in presence of DFT+U corrections. The values for U are reported in Table S1.

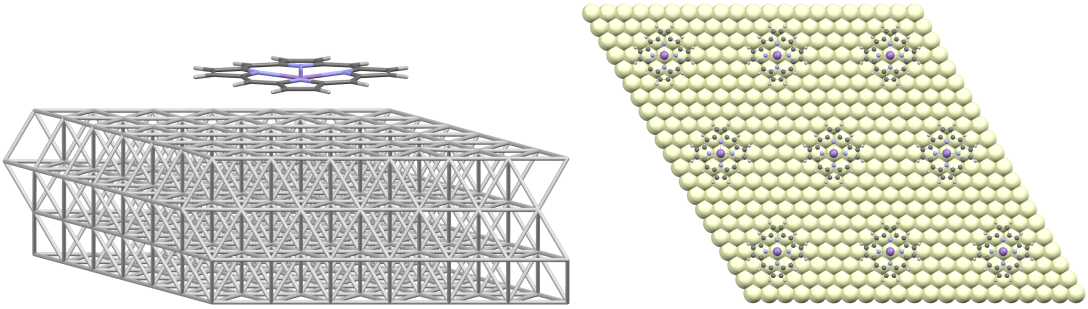

Figure S2: Left: 3D representation of TMPP adsorbed on Ag(111) used in our calculations. Right: a 3×3 supercell representation of the geometric model used in our calculations; it can be seen that the separation between periodically repeated images of the molecules is large enough to minimize the lateral interactions.

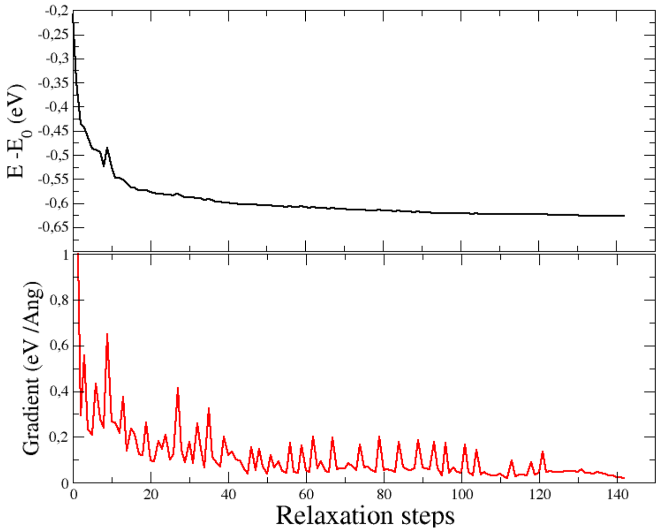

Figure S3: Dependence of energy (top) and gradient (bottom) on the step of the conjugate-gradient algorithm for structural relaxation; VPP system, "bridge" position. A constant quantity, E_0 was removed from all energy values. We note a very slow decrease of the energy as a function of relaxation step from a gradient ≈ 0.02 eV/Å .

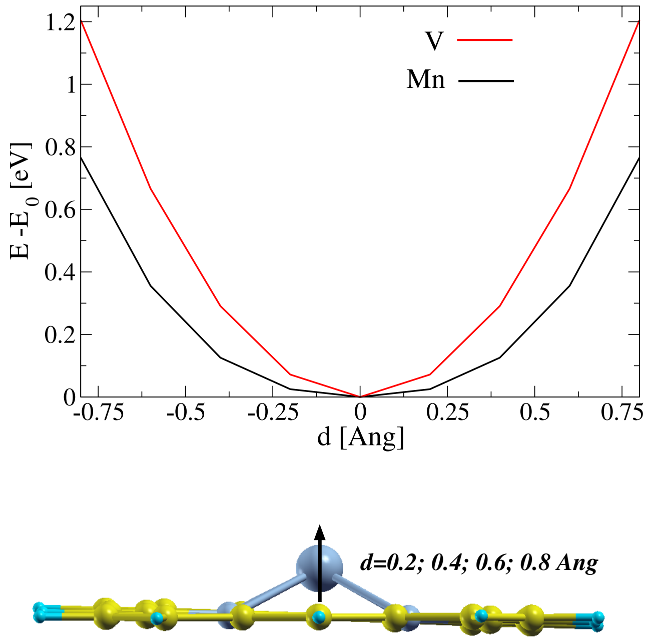

Figure S4: Dependence of total energy in VPP and MNPP on the distance between center-of-mass of the relaxed molecule and the TM (TM=V, Mn). In each case, the reference (structure at $d = 0$) was the relaxed molecule.

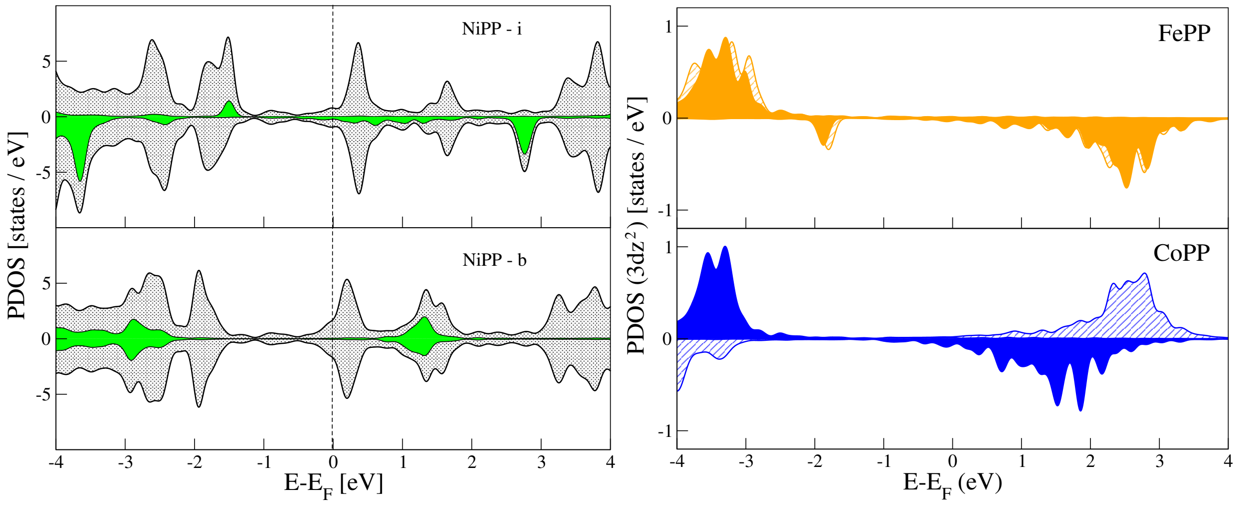

Figure S5: Left: Density of states of NiPP in the "intermediate" and "bridge" points. Grey - whole molecule; green - only Ni atom. Right: PDOS for the $3dz^2$ states in the CoPP and FePP, respectively. We see that for the FePP no differences occur between "t" (hatch) and "b" (filled) sites, while the CoPP is more sensitive to the adsorption site.

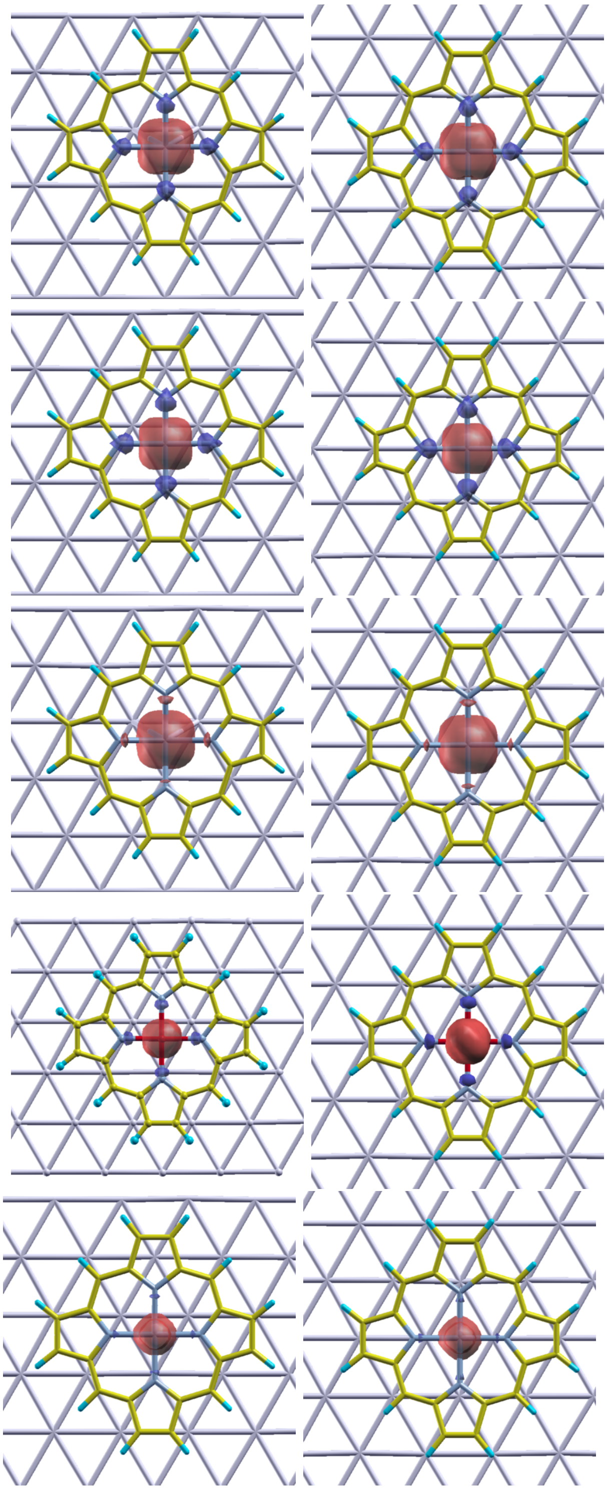

Figure S6: Spatial representation of the spin density for TMPP adsorbed in hollow (left) and bridge positions (right). From top to bottom, VPP, CrPP, MnPP FePP and CoPP. Contour plots are represented for a density of 0.005 spins/Bohr ^{3}; this represents $\approx$ 1 % of the maximum value of the spin density in all cases (i.e. around 0.5 spins/Bohr ^{3}). For MnPP the representation is done at 0.003 spins/Bohr ^{3} which is close to the maximum absolute value for spin-down. We discard the data for NiPP, where the magnetic moment is close to zero.

Table S4: The most important relative weight of the PDOS for selected orbitals in the molecule (contribution of silver atoms was discarded). We use the symbol $\mathbf{2p_z}$ to indicate the sum of contributions delocalized orbitals over all N and C atoms, respectively (i.e. delocalized π state). Orbital energies are relative to Fermi level. By σ we symbolize the spin orientation; up and down are represented by corresponding arrows.

MPP	σ	E (eV)	Orbital
VPP	↑	-1.44	$3d_{(x^2-y^2)}$ on V (82 %) + $\mathbf{2p_z}$
	↑	-1.08	$3d_{xz}$ on V (28 %) + $3d_{yz}$ on V (27 %) + $\mathbf{2p_z}$
	↑	0.34	$3d_{z^2}$ on V (10 %) + $4s$ on V (5 %) + $3d_{yx}$ on V (3 %) + $\mathbf{2p_z}$
	↓	-1.77	$2p_z$
	↓	0.14	$2p_z$
CrPP	↑	-3.18	$3d_{(x^2-y^2)}$ on Cr (51 %) + $3d_{yz}$ on Cr (7 %) + $3d_{xz}$ on Cr (3 %) + $\mathbf{2p_z}$
	↑	-2.01	$3d_{yz}$ on Cr (10 %) + $3d_{xz}$ on Cr (10 %) + $3d_{yz}$ on Cr (4 %) +$\mathbf{2p_z}$
	↑	0.27	$\mathbf{2p_z}$
	↓	-1.93	$\mathbf{2p_z}$
	↓	0.11	$\mathbf{2p_z}$
MnPP	↑	-1.89	$\mathbf{2p_z}$
	↑	-0.56	$3d_{xy}$ on Mn (40 %) + $2s$ on N (12 %) + $2p_x$ and $2p_y$ on N
	↑	0.29	$\mathbf{2p_z}$
	↓	-1.73	$\mathbf{2p_z}$
	↓	0.20	$\mathbf{2p_z}$
FePP	↑	-3.05	$3d_{(x^2-y^2)}$ on Fe (50 %) + $\mathbf{2p_z}$
	↑	-1.90	$\mathbf{2p_z}$
	↑	0.26	$\mathbf{2p_z}$
	↓	-1.08	$3d_{yz}$ on Fe (30 %) + $3d_{xz}$ on Fe (50 %) + $\mathbf{2p_z}$
	↓	0.29	$3d_{yz}$ on Fe (4 %) + $3d_{xz}$ on Fe (3 %) + $\mathbf{2p_z}$
CoPP	↑	-2.30	$3d_{yz}$ on Co (20 %) + $3d_{xz}$ on Co (18 %) + $\mathbf{2p_z}$
	↑	-1.90	$\mathbf{2p_z}$
	↑	0.25	$\mathbf{2p_z}$
	↓	-1.94	$3d_{yz}$ on Co (17 %) + $3d_{xz}$ on Co (15 %)
	↓	0.26	$\mathbf{2p_z}$
NiPP-h	↑	-2.60	$3d_{yz}$ on Ni (4 %) + $3d_{z^2}$ on Ni (3 %) + $3d_{xz}$ on Ni (4 %) + $\mathbf{2p_z}$
	↑	-1.92	$\mathbf{2p_z}$
	↑	0.20	$\mathbf{2p_z}$
	↓	-1.96	$\mathbf{2p_z}$
	↓	0.18	$\mathbf{2p_z}$
NiPP-i	↑	-1.82	$\mathbf{2p_z}$
	↑	-1.55	$3d_{xy}$ on Ni (17 %) + $2s$ on N (10 %) + $\mathbf{2p_z}$
	↑	0.34	$\mathbf{2p_z}$
	↓	-1.82	$\mathbf{2p_z}$ on C
	↓	0.34	$4s$ on Ni (4%) + $3d_{z^2}$ on Ni (9 %) + $\mathbf{2p_z}$
